# Socioeconomic inequality in dietary intake begins before 24 months in Brazilian children

**DOI:** 10.11606/S1518-8787.2019053000679

**Published:** 2019-01-18

**Authors:** Ana Elisa Madalena Rinaldi, Wolney Lisboa Conde

**Affiliations:** I Universidade Federal de Uberlândia. Faculdade de Medicina. Curso de Nutrição. Uberlândia, MG, Brasil; II Universidade de São Paulo. Faculdade de Saúde Pública. Departamento de Nutrição. São Paulo, SP, Brasil

**Keywords:** Infant Nutrition, Breast Feeding, Infant Food, Diet, Food, and Nutrition, Feeding Behavior, Diet Surveys, Dietary Patterns

## Abstract

**OBJECTIVE:**

To assess dietary patterns by socioeconomic gradient of Brazilian infants and young children in 2006 and 2013.

**METHODS:**

Data from the National Demographic Survey (2006) and the National Health Survey (2013) were used. Food intake were described by wealth index, age range and survey year. Dietary patterns were defined by principal component analysis. Association of wealth index and dietary patterns were modelled using linear regression.

**RESULTS:**

Breast milk intake was higher for poor infants and young children, while fresh food intake (fruits, vegetables, meats, beans) was higher for the richer ones in 2006 and 2013. Biscuits and sweetened beverages were more consumed by rich infants and young children in 2006 and by poor and rich children in 2013. Three dietary patterns (DP1, DP2, and DP3) were identified in 2006 and four in 2013 (DP1, DP2, DP3, and DP4). DP1 was composed mainly of fresh foods, and it was positively associated with the wealth index for infants and young children in both years. DP2 was composed of biscuits, cookies and sweetened beverages, and it was positively associated with the wealth index for young children in 2006 and for poor and rich infants and young children in 2013. DP3 was composed of milk, water and porridge in both years, and it was not associated with the wealth index. DP4 was composed of breast milk and porridge, and it was negatively associated with the wealth index.

**CONCLUSIONS:**

DP1 is a characteristic pattern for richer infants and young children since 2006, while DP2 is a characteristic pattern for all infants and young children in 2013, regardless of wealth index. Dietary inequality between the poor and the rich seems to begin in childhood.

## INTRODUCTION

As the first 1,000 days of life is considered a window of opportunity for achieving optimum growth and long-term health benefits^1^, feeding practices can play an important role in this period. Infants will be more willing to try new foods if they are breastfed and exposed to a large variety of tastes and textures at an early age^2^. Once dietary patterns are defined and established in infancy, it is more difficult to change them in the future^3^ ^,^ ^4^. In this period of life, the infants and young children develop their food practices and preferences, defined by the cultural, social, and economic context, in which the family is inserted.

The family context, especially socioeconomic factors, defines dietary patterns in childhood. The tracking of dietary patterns from six to 12 months of life was more associated with maternal sociodemographic factors than with infant growth. Higher maternal schooling was associated with healthier transition from breastfeeding to dietary guidelines^4^. In different studies, dietary patterns labeling “healthy” or “guidelines” or “breastfeeding” or “fruits, vegetables and high-protein foods” during infancy were positively associated with higher maternal education and higher income^3^ ^,^ ^5^ ^-^ ^8^.

In Brazil, high-quality and diversified diets were available only to 28% of infants and 20% of toddlers in 2006. This diet is 40% less probable among poor children than among richer ones^9^. The results from the last national survey developed in Brazil (2013) identified that 32% of children under two years consume sugary drinks. Also, the consumption of this kind of beverage was not associated with family schooling, but with the children's family consumption^10^. Social inequality in the diet of infant and young children is an emerging issue in the Brazilian dietary guidelines.

Infant feeding is mainly influenced by feeding practices from the family, and results from secular trend analysis (1987 to 2008) about dietary intake from household budget surveys have been marked by two negative aspects, mainly for adult members: a decrease from 44.0% to 38.9% of total daily energy intake of unprocessed foods and an increase from 18.7% to 29.6% of total daily energy intake of ultra-processed foods^11^. Also, the increase in ultra-processed foods occurred in all income quintiles. Therefore, it is relevant to analyze how the feeding of infants and young children are influenced by the family dietary intake. As we believe the description of feeding practices from 2000 to 2010 has not been published in Brazil, it is relevant to assess the current feeding practices to determine if they are affected by income in childhood as they are in adulthood. Therefore, our objective was to assess dietary patterns by socioeconomic gradient of Brazilian infants and young children under 24 months between 2006 and 2013.

## METHODS

### Data Source

Data from two Brazilian National Surveys carried out in this century were used. The first one is the National Demographic and Health Survey of Children and Women (PNDS) between 2006–2007^12^. This kind of survey covers the population of women during their reproductive age and children under five years old. The other one is the National Health Survey (PNS) from 2013, whose goal is to describe the health status and lifestyles of Brazilian adults and older adults, their access to healthcare services, preventive actions, and health assistance funding^13^.

### Study Design and Population

The sampling of both national household-based surveys involve complex probabilistic sampling design with clustering and stratification. The PNDS sample was stratified by geographic regions and constructed in two stages. The primary and secondary sample units were clusters and households, respectively. Each woman in reproductive age and her children under five years were selected in each household. The PNS sample was selected by clustering primary sampling units within three stages obtained after household social and regional stratification. In short, the first stage was the selection of the primary sampling units, the second was the selection of households, and the third was the selection of adult residents to answer the questionnaire^14^. Although the PNS surveyed adults and older adults, a brief section of the questionnaire gathered data about the health of infants and young children younger than 24 months of age. Children were selected in the second stage, similar to PNDS.

The sample assigned for this study included infants and young children younger than 24 months of age, who were alive on the interview date and living with their mother or legal guardians. After applying these criteria, 1,904 children (96.5% of total children in 2006–2007) and 4,215 (81.0% of total children in 2013) were included in the final sample. In 2013, only the youngest child of the family was selected.

### Dietary Intake

The dietary intake in both surveys was assessed using a questionnaire about foods offered in the previous day (current status data) with yes-no questions. Almost all foods were the same in both surveys. The food items in PNDS (2006–2007) were: breast milk, milk and formula, water, tea, porridge, rice or pasta, bread, beans, tubers, greens, vegetables, fruit, fruit juice, red meat or pork, liver, chicken, fish, eggs, fried foods, sweets (candies, chocolate and other foods with sugar), biscuits, homemade snacks, chips, yogurt and soda or industrialized (sweetened) juice. The food items in PNS (2013) were: breast milk, milk and formula, water, tea, porridge, cereals (rice, pasta, bread), beans, tubers, vegetables or greens, fruit/fruit juice, meat (all types) or eggs, sweets (candies, chocolate and other foods with sugar), biscuits, soda, industrialized (sweetened) juice, and other foods (not described).

In PNDS, some foods were aggregated to be comparable with those in PNS, such as: rice and bread (cereals); fruits and fruit juices; and all types of meat and eggs (red meat, chicken, fish, pork, liver). In this study, missing values or “I do not know” answers for any food items were replaced by 0 (0 = no), i.e. the child had not consumed that specific food in the previous 24 hours as proposed by the World Health Organization (WHO)^15^. The percentage of replaced values was 2.0% in PNDS and 0% in PNS.

### Demographic Variable

All analysis were performed by age range. The first range was composed of infants (zero to 11.9 months) and young children (12 to 23.9 months). In PNS (2013), the age of children was available only as rounded-up values: 0 (zero to 11.9 months) and 1 (12 to 23.9 months). In PNDS, the age was rounded up and categorized in a similar way.

### Socioeconomic Variable

The wealth index was applied as a proxy for income, as recommended by Filmer and Pritchett^16^ for the Demographic Health Surveys Program. In PNDS (2006), the information about income was unavailable. Principal component analysis was applied, and the score of the first component in both surveys was selected. The first component was used to predict the scores assigned to each child, then, the scores were divided into quintiles.

The wealth index was calculated using information about the assets available on households, sewage disposal facility, and schooling of the breadwinner. In PNDS, the items selected to calculate the wealth index were toilet facility, bathroom, television, fridge, VCR player, washing machine, telephone, car, computer, housemaid, vacuum cleaner, and schooling of the breadwinner. In PNS, the items were bathroom, television, fridge, DVD player, washing machine, telephone, car, computer, housemaid, cell phone, microwave, motorcycle, and internet. The first component was defined using eigenvalues higher than 1.0 and characterized by variables with eigenvectors higher than 0.2. The Kaiser-Meyer-Olkin (KMO), which analyzes the compliance of variables to PCA, was 0.91.

### Statistical Analysis

The prevalence of each food item was described by the 1^st^ (the lowest wealth index quintile – the poorest infants and young children) and 5^th^ (the highest wealth index quintile – the richest infants and young children) wealth index quintiles, age range, and survey year. The Wald tests were applied to compare the prevalence between 1^st^ and 5^th^ quintiles.

The dietary patterns were defined by principal component analysis adopting eigenvalues higher than 1.0 and eigenvectors (loadings) higher than 0.2. The dietary patterns were defined for PNDS (2006–2007) and for PNS (2013) separately, because some foods were different. In each survey, all food items available in the questionnaire were maintained. For practical reasons, in PNS (2013), “other foods” were excluded, because it was not possible to define what kind of food was included in that category, and the frequency of this food item was only 2.8%. Foods with low intake frequency were excluded in other studies for similar reasons^6^ ^,^ ^7^ ^,^ ^17^. After dietary pattern extraction, an orthogonal rotation (Varimax) was applied to improve dietary pattern interpretation. We used Kaiser-Meyer-Olkin (KMO) to analyze the compliance of variables with the principal component analysis. Each child received a standardized score for each dietary pattern identified. The value of the score represents the closeness of the child to each dietary pattern. The mean scores of each dietary pattern was plotted by the 1^st^ and 5^th^ wealth index quintiles by age range during survey years. Linear regression was applied to compare the dietary pattern scores in the 1^st^ and 5^th^ quintiles within age range and survey year. The sample weight was considered in all analysis, which were carried out with Stata/MP 13.0®.

## RESULTS

The number of eligible infants and young children was 1,904 and 4,215 in 2006 and 2013, respectively. The proportion of infants was 53.6% and 46.4% in 2006 and 2013, respectively, and the proportion of young child aged 12 to 24 months was 50.1% and 49.9% in 2006 and 2013, respectively.

The prevalence of breast milk intake decreased from 76.0% to 34.4% for young children in 2006–2007 and from 69.7% to 35.9% in 2013 (data not tabulated). The prevalence of breastfeeding was higher in the 1^st^ wealth index quintile in 2006 and 2013, except for infants in 2006. In 2006, the gap favorable to poor infants and to young children was 12.9 and 25.1 percentage points, respectively; in 2013, it was 13.8 and 19.5 percentage points, respectively.

Table 1 shows the food consumption of infants and young children during both survey years and for the 1^st^ and 5^th^ quintiles. The percentages of all foods were higher for young children in both years. Most fresh foods (fruits, vegetables, beans, meats, tubers) prevailed among infants and young children in the 5^th^ wealth index quintile (the richest one) in both years. In 2006, industrialized juices and soda, fried foods and homemade snacks prevailed among rich young children, while breastmilk was more frequent among the poor one. In 2013, breastmilk, industrialized juices and soda prevailed among infants and young children in the 1^st^ wealth index quintile (the poorest one).

**Table 1:** Prevalence of food intake (%) of infants and young children according to the 1^st^ and 5^th^ wealth index quintiles and age range by survey. Brazil, PNDS (2006) and PNS (2013).

Variable	Age range					
	0–11 months			12–23 months		
	Wealth index (poorest and richest)					
	1^st^	5^th^	Relative inequality (5^th^ *versus* 1^st^)	1^st^	5^th^	Relative inequality (5^th^ *versus* 1^st^)
Food items^a^/Survey year			p			p
2006/2007						
Breast milk	83.6	70.7	0.0894	49.4	24.3	0.0014
Milk	64.5	56.9	0.3913	76.8	82.4	0.4384
Water	81.1	68.2	0.0803	87.5	71.2	0.0009
Tea	27.5	12.9	0.0714	10.6	16.5	0.4334
Porridge	41.8	42.5	0.9388	48.7	54.2	0.5211
Fruits and fruit juice	45.9	65.0	0.0348	88.0	100.0	0.0000
Vegetables	26.5	56.6	0.0007	62.2	93.1	0.0000
Beans	31.2	47.4	0.0692	85.7	96.9	0.0007
Meats	27.3	52.3	0.0039	94.0	99.1	0.0096
Tubers	24.4	58.0	0.0001	49.2	91.4	0.0000
Cereals	38.8	48.9	0.2673	96.3	100.0	0.0019
Biscuits, cookies and cake	27.5	46.6	0.0253	88.8	94.7	0.1560
Sweets	13.5	19.7	0.4134	64.3	62.8	0.8687
Industrialized juice and soda	16.7	13.0	0.5718	48.5	76.4	0.0001
Fried foods^b^	7.5	11.5	0.5714	27.6	53.1	0.0023
Chips^b^	14.1	10.0	0.5709	57.5	42.2	0.0667
Homemade snacks^b^	0.4	9.1	0.1031	14.9	29.4	0.0037
Yogurt^b^	22.7	38.9	0.0524	53.3	87.3	0.0000
2013						
Breast milk	77.4	63.6	0.0111	46.5	27.0	0.0006
Milk	56.5	63.3	0.2368	76.8	91.5	0.0001
Water	87.5	68.2	0.0022	99.3	96.7	0.1125
Tea	16.0	17.5	0.7034	17.6	11.4	0.1296
Porridge	53.0	20.4	0.0000	52.9	30.5	0.0001
Fruits and fruit juice	44.1	60.4	0.0046	74.8	94.9	0.0000
Vegetables	34.0	50.1	0.0052	52.3	92.2	0.0000
Beans	33.2	44.7	0.0412	78.3	91.2	0.0001
Meats	27.6	41.9	0.0086	74.1	89.9	0.0001
Tubers	24.2	46.3	0.0001	38.1	74.0	0.0000
Cereals	38.4	45.3	0.2256	80.8	86.7	0.2220
Biscuits, cookies and cake	38.5	36.2	0.6762	71.4	82.1	0.0350
Sweets	13.3	7.8	0.0559	40.9	46.6	0.3702
Industrialized juice^c^	12.5	6.3	0.0347	39.2	37.0	0.7145
Soda^c^	5.0	2.3	0.1511	25.4	23.1	0.6657
Industrialized juice and soda	19.8	8.4	0.0104	54.5	47.9	0.3698

a The frequencies of food items represent only children who answered “yes.”

b These variables were available only in 2006.

c These variables was available only in 2013.

Three dietary patterns were defined in 2006 and four in 2013, and these dietary patterns represent more than 50% of the variance. KMO was high in both years. The first dietary pattern (DP1) was composed almost exclusively of fresh foods with the exception of biscuits, cookies and cakes, and the values of eigenvectors (loadings) were similar in both survey years; the second dietary pattern (DP2), by industrialized foods in both years; the third (DP3), by milk and other semi-solid foods in both years; and the fourth (DP4), by breast milk and semi-solid foods (Table 2).

**Table 2:** Dietary patterns (DP) for infants and young children by each survey year. Brazil, PNDS (2006) and PNS (2013).

Variable	Survey year						
	2006			2013			
	DP1	DP2	DP3	DP1	DP2	DP3	DP4
Breast milk			-0.4247			-0.5654	0.2286
Milk (non breast milk)			0.5167			0.6133	
Water	0.2057		0.3056			0.2726	
Tea	-0.2376		0.4878				0.8268
Porridge			0.4423			0.4350	0.3866
Fruits and fruit juices	0.3306			0.3526			
Vegetables	0.3416			0.4553			
Beans	0.3421			0.3705			
Meat	0.3334			0.3404			
Tubers	0.3169			0.4284			
Cereals	0.3516			0.3482			
Biscuits, cookies and cake	0.3238			0.2512	0.2077		
Sweetened foods		0.3893			0.5033		
Sweetened beverages^a^		0.3469					
Fried foods^b^		0.5335					
Chips^b^		0.4446					
Homemade snacks^b^		0.4319					
Yogurt^b^	0.2838						
Industrialized (sweetened) juices^c^					0.5134		
Soda^c^					0.5941		
Variance (%)	59.2			58.8			
KMO	0.9479			0.9028			

KMO: Kaiser-Meyer-Olkin

a Sweetened beverages: industrialized (sweetened) juice and soda.

b These variables were available only in 2006.

c These variables were available only in 2013.

In 2006, the DP1 score was higher for infants and young children in the 5^th^ wealth index quintile, while the DP2 was higher for young children in the same quintile (Figure A, Table 3). In 2013, the DP1 score was also higher for infants and young children in the 5^th^ wealth index quintile. The DP2 score was similar for all age range and wealth index quintiles. The DP4 score was higher for infants and young children in the 1^st^ wealth index quintile (Figure B, Table 4).

**Figure:**
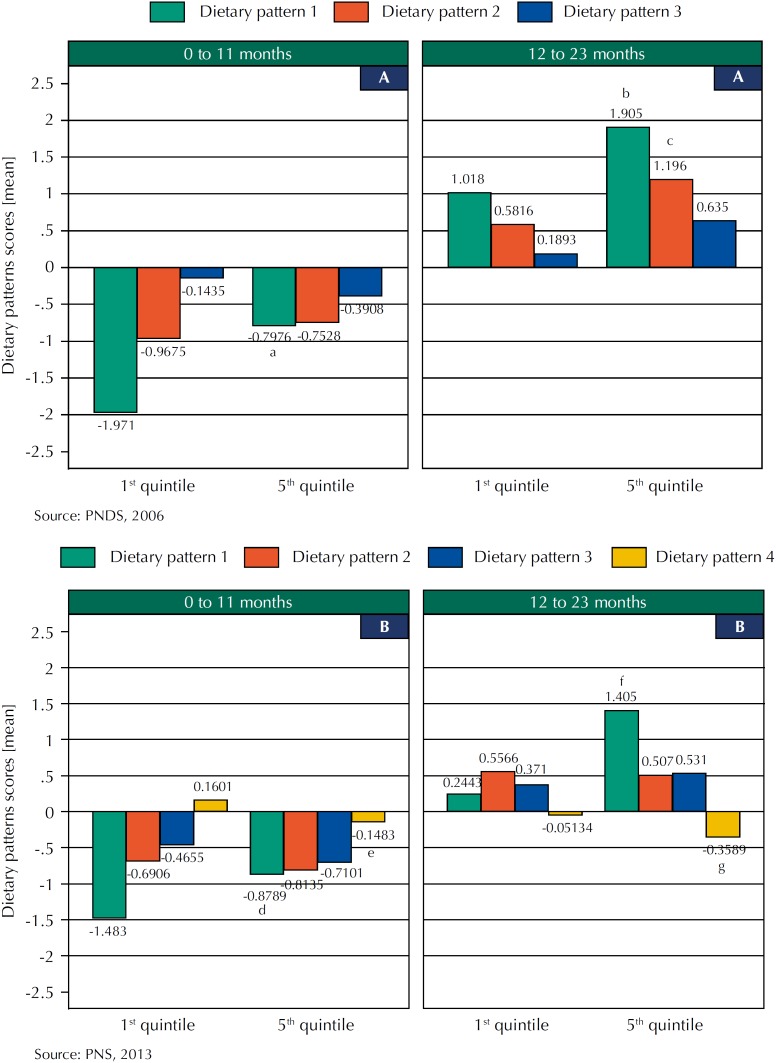
Means of dietary pattern scores by the poorest (1^st^) and richest (5^th^) wealth index and age range in 2006 (A) and 2013 (B). Brazil, PNDS (2006) and PNS (2013). Comparison between 1^st^ quintile *versus* 5^th^ quintile by age range: ^a^p = 0.016; ^b^p = 0.000; ^c^p = 0.012; ^d^p = 0.023; ^e^p = 0.000; ^f^p = 0.003; ^g^p = 0.005.

**Table 3:** Linear regression coefficients of dietary patterns according to socioeconomic gradients by age range. Brazil, PNDS (2006).

Wealth index	Dietary pattern 1		Dietary pattern 2		Dietary pattern 3	
	0–11 months	12–23 months	0–11 months	12–23 months	0–11 months	12–23 months
*β* (95%CI)						
1^st^ quintile	Reference	Reference	Reference	Reference	Reference	Reference
2^nd^ quintile	0.54(-0.30–1.38)	0.64(0.34–0.95)	0.37(-0.17–0.90)	0.00(-0.32–0.33)	-0.32(-0.85–0.22)	0.11(-0.19–0.42)
3^rd^ quintile	0.21(-0.69–1.10)	0.75(0.48–1.03)	0.18(-0.22–0.57)	0.62(0.24–0.99)	-0.19(-0.68–0.31)	0.32(-0.38–0.67)
4^th^ quintile	0.87(-0.10–1.84)	1.00(0.73–1.26)	0.11(-0.29–0.50)	0.52(-0.06–1.10)	-0.41(-0.92–0.10)	0.38(0.10–0.66)
5^th^ quintile	1.17(0.21–2.13)	0.89(0.64–1.14)	0.21(-0.39–0.82)	0.61(0.13–1.09)	-0.25(-0.72–0.22)	0.45(-0.03–0.92)

**Table 4:** Linear regression coefficients of dietary patterns according to socioeconomic gradients by age range. Brazil, PNS (2013).

Wealth index	Dietary pattern 1		Dietary pattern 2		Dietary pattern 3		Dietary pattern 4	
	0–11 months	12–23 months	0–11 months	12–23 months	0–11 months	12–23 months	0–11 months	12–23 months
*β* (95%CI)								
1^st^ quintile	Reference	Reference	Reference	Reference	Reference	Reference	Reference	Reference
2^nd^ quintile	0.53 (0.15–0.90)	0.52 (0.27–0.77)	0.09 (-0.08–0.25)	0.24 (0.00–0.47)	-0.00 (-0.26–0.26)	0.08 (-0.13–0.29)	-0.00 (-0.19–0.18)	-0.18 (-0.34– -0.02)
3^rd^ quintile	1.20 (0.79–1.62)	0.92 (0.70–1.14)	0.23 (-0.02–0.48)	0.19 (-0.10–0.47)	0.07 (-0.21–0.35)	0.17 (-0.06–0.40)	0.10 (-0.13–0.33)	0.08 (-0.18–0.33)
4^th^ quintile	1.22 (0.79–1.64)	1.05 (0.80–1.30)	-0.08 (-0.25–0.08)	0.07 (-0.20–0.34)	-0.04 (-0.31–0.22)	0.31 (0.10–0.51)	0.06 (-0.16–0.28)	-0.14 (-0.35–0.08)
5^th^ quintile	0.60 (0.08–1.12)	1.16 (0.91–1.41)	-0.12 (-0.27–0.03)	-0.05 (-0,41–0.31)	-0.24 (-0.57–0.08)	0.16 (-0.07–0.39)	-0.31 (-0.51– -0.10)	-0.31 (-0.52– -0.09)

In our study, we analyzed if there is socioeconomic inequality among Brazilian infants and young children and if this scenario changed in a gap of seven years (2006 and 2013). In 2006, the gap between infants and young children in the 1^st^ and 5^th^ wealth index quintiles for DP1 was +1.17 and +0.89 scores, respectively; and the gap for young children for DP2 was +0.61. According to the results, inequality was greater among infants in 2006 (Figure A). In 2013, the gap between infants and young children in the 1^st^ and 5^th^ wealth index quintiles for DP1 was +0.60 and +1.16 scores, respectively; and for DP4 was −0.02 and −0.31, respectively. In 2013, the gap between infants in the 1^st^ and 5^th^ wealth index quintiles decreased, but, based on differences in DP1, it increased among young infants.

## DISCUSSION

We highlighted three main results of our study. DP1 is characterized mainly by fresh foods and is more likely among rich infants and young children in both decades. DP2, on the other hand, is characterized mainly by industrialized foods with high sugar content, is the second dietary pattern in both years, and its wealth index increased only for young children in 2006; in 2013, the DP2 scores were similar between poor and rich infants and young children. Also, the breastfeeding practices in Brazil are still more common among poor infants and young children.

In our study, breastfeeding prevailed among poor infants and young children either as isolated practices (Table 1) or with other foods in dietary patterns (Table 4). DP4 occurred only in 2013, and the scores were higher among poor infants and young children. A recent study^18^ involving developed and developing countries described that the breastfeeding duration is negatively associated with the income of a country. There was an inverse association between breastfeeding at six months and log gross domestic product *per capita*. The authors estimated that breastfeeding prevalence at 12 months decreased by 10 percentage points for each doubling in the gross domestic product *per capita* ^18^. The breastfeeding practice has increased in Brazil in the last three decades in response to many promotions, support, and protective actions^19^. The prevalence of continued breastfeeding up to two years of age increased from 24.5% to 31.8% between 1986 and 2013^20^. This increase was accompanied by extended breastfeeding. The median duration of breastfeeding increased from 2.5 months in 1974 to 7.0 months in 1996, reaching 14.0 months in 2006^19^.

Unlike breastfeeding, the consumption of fresh foods prevails among richer infants and young children since 2006, when they were assessed isolated or as a component in dietary patterns. Although Brazil has experienced a decrease in maternal and child health care inequities, the socioeconomic inequities impact the diet quality of the poor^20^. Higher wealth index increases the accessibility to this kind of food and the tools available to find dietary guidelines for children. All previous studies developed in Brazil and in other countries found positive association between wealth index and dietary patterns composed of fresh or healthy foods^6^ ^,^ ^9^ ^,^ ^17^ ^,^ ^21^. In general, dietary patterns labelled healthy, healthy conscious or guidelines have been positively associated with the frequency of breastfeeding, maternal education, and highest quintile of wealth^5^ ^,^ ^6^ ^,^ ^8^ ^,^ ^22^.

DP2 was composed of industrialized foods, mainly the sweetened ones, such as biscuits, industrialized juices and soda, in 2006 and 2013. This dietary pattern prevailed among rich young children in 2006. However, the protective effect for the poor disappeared because no difference was found between poor and rich infants and young children in 2013. Richer infants and young children are more protected from industrialized foods because the DP1 score was higher. The prevalence of these foods, especially biscuits, were higher than 20% among infants and young children regardless of the wealth index. The prevalence of this kind of food doubled after 12 months of age, which is worrisome because this food contains high amount of sugar, fat and artificial chemical components, and this period of life is critical to develop the taste. We noticed that in all studies there is at least one dietary pattern composed of ultra-processed foods with soda, industrialized juices and sweetened foods. In a follow-up of a birth cohort in the Netherlands, the dietary pattern labelled as western-like, which was characterized by ultra-processed foods, was associated with unfavorable family lifestyle and low socioeconomic background^23^. In Spain, the dietary pattern characterized by biscuits, sweets and crisps was associated with low educational level, more children and younger maternal age^5^.

The presence of industrialized foods, mainly biscuits, industrialized juices and soda in dietary patterns of infants and young children reflects the dietary patterns from the home environment. The intake of sweetened beverages by Brazilian infants and young children was association with their parent's intake of this food^10^. Although maternal or parental education affects the children's food consumption, this relationship was mediated by the mother's diet^24^. Brazil is facing changes in dietary patterns following those seen in developed countries such as Canada and Britain^25^ ^,^ ^26^, where the consumption of ultra-processed foods has been increasing and that of unprocessed foods has been decreasing. Although these national and international studies did not assess specific data on the feeding practices of infants and young children, they are important to understand what kinds of foods are purchased by adults and consequently available for all household members. This movement could be changed with income distribution, social transformations and a strong presence of the food industry in the country^11^ ^,^ ^27^. The price per calories of some industrialized or ultra-processed foods is cheaper than fresh foods, mainly fruits and vegetables. However, unprocessed or minimal processed foods, such as beans, rice and chicken prepared at home could be cheaper than replacing homemade meals for ultra-processed foods^28^.

With this study, we updated the characteristics of infants and young children's dietary patterns in 2013 and analyzed them by wealth health. The national data about the feeding practices of infants and young children is restricted to Brazilian demographic health surveys (1986, 1996, and 2006). Although the population study from PNS was not infants and young children, it shows some data for these age group. Almost all questions about infant feeding practices were equal for PNS and PNDS (2006). Another strength of our study is the description of Brazilian infants and young children feeding practices as dietary patterns. This multivariate analysis approach allowed us to summarize the main characteristics of feeding as a whole dimension to highlight the early socioeconomic influence and to trace their trends.

Our study has some important limitations because of the data source. The first one is the differences among food items in the questionnaires from 2006 and 2013. Dietary patterns can be altered when the foods are different, as the aim of the analysis is to extract the main characteristics from a data pool. However, we understood that this difference was not relevant because the composition of dietary pattern was very similar. The main difference from 2006 to 2013 was DP4, which was composed mainly of breastmilk, tea and porridge [eigenvectors (loadings) > 0.2]. In 2006, breastfeeding had a negative loading in DP3, and tea and porridge were also present in this dietary pattern. The other limitation is the format of food data (yes-no answers), because it inhibited the assess to the amount of food consumption. However, the feeding practices of infants and young children in national surveys are traditionally assessed by the presence or absence on the previous day, as recommended by the World Health Organization^15^. Principal component analysis is usually applied for quantitative variables but, when the purpose of the study is to describe dietary patterns, it was possible to apply for binary variables^29^. Another limitation is the age provided in completed years in PNS (2013). It was only possible to know if the child was aged under or above 12 months. Because of this, we could not to separate the analysis for infants under 6 months nor assess the early introduction of industrialized foods or other foods not appropriate for this age range. The last limitation concerns the secular trend of dietary pattern between 2006 and 2013. We analyzed the dietary patterns by age range and wealth index for each survey year separately, because PNDS and PNS have methodological differences that can affect the comparison.

The inequality between the feeding practices of poor and rich children seems to begin in infanthood. Higher wealth index predicts higher scores for dietary patterns composed mainly of fresh foods (DP1) between 2006 and 2013. The consumption of industrialized foods, mainly sweetened foods, is present in the dietary patterns of poor and rich infants and young children. Breastfeeding is the only pro-poor feeding practice in Brazil. We expect Brazilian Breastfeeding and Complementary Feeding Strategy to play an important role to stimulate the increase of the frequency and duration of exclusive breastfeeding and decrease the consumption of ultra-processed foods by infants and young children. Also, this important health strategy needs to be accompanied by economic policies to decrease the price of fresh foods.
